# Monitoring Hydroxycinnamic Acid Decarboxylation by Lactic Acid Bacteria Using High-Throughput UV-Vis Spectroscopy

**DOI:** 10.3390/molecules25143142

**Published:** 2020-07-09

**Authors:** Gonzalo Miyagusuku-Cruzado, Israel García-Cano, Diana Rocha-Mendoza, Rafael Jiménez-Flores, M. Monica Giusti

**Affiliations:** Department of Food Science and Technology, The Ohio State University, 2015 Fyffe Ct., Columbus, OH 43210-1007, USA; miyagusukucruzado.1@osu.edu (G.M.-C.); garciacano.1@osu.edu (I.G.-C.); rochamendoza.1@osu.edu (D.R.-M.); jimenez-flores.1@osu.edu (R.J.-F.)

**Keywords:** *p*-hydroxystyrene, 3,4-dihydroxystyrene, 4-hidroxy-3-mthoxystyrene, flavor compounds, lactic acid bacteria

## Abstract

Hydroxycinnamic acid (HCA) decarboxylation by lactic acid bacteria (LAB) results in the production of 4-vinylplenols with great impact on the sensorial characteristics of foods. The determination of LAB decarboxylating capabilities is key for optimal strain selection for food production. The activity of LAB strains from the Ohio State University—Parker Endowed Chair (OSU-PECh) collection potentially capable of synthesizing phenolic acid decarboxylase was evaluated after incubation with HCAs for 36 h at 32 °C. A high-throughput method for monitoring HCAs decarboxylation was developed based on hypsochromic shifts at pH 1.0. Out of 22 strains evaluated, only *Enterococcus mundtii*, *Lactobacillus plantarum* and *Pediococcus pentosaceus* were capable of decarboxylating all *p*-coumaric, caffeic and ferulic acids. Other strains only decarboxylated *p*-coumaric and caffeic acid (6), only *p*-coumaric acid (2) or only caffeic acid (1), while 10 strains did not decarboxylate any HCA. *p*-Coumaric acid had the highest conversion efficiency, followed by caffeic acid and lastly ferulic acid. Results were confirmed by HPLC-DAD-ESI-MS analyses, showing the conversion of HCAs into their 4-vinylphenol derivatives. This work can help improve the sensory characteristics of HCA-rich foods where fermentation with LAB was used during processing.

## 1. Introduction

Hydroxycinnamic acids (HCAs) are secondary metabolites in plants and fungi characterized by a C6–C3 structure [1]. At high concentrations, these compounds exert an antibacterial effect over a wide range of Gram-positive and Gram-negative bacteria [2,3,4]. Further studies have shown that some bacterial strains can decarboxylate HCAs as a mechanism for detoxification of its environment, given that the antimicrobial activity of the aforementioned acids depends on the presence of a double bond in the side chain of the structure [5]. However, it also has been reported that not all bacterial [6] or all yeast strains [7] are capable of decarboxylating HCAs that lead to the formation of 4-vinylphenols (4VPs) or their reduced form, 4-ethylphenols. In fermented products such as wine, HCA degradation by yeast results in the formation of 4VPs; which are responsible for imparting distinct phenolic off-flavors in the final product [7]. However, depending on the application, these compounds may be desirable for cases such as wheat beer, where the reduction in 4VP and 4-vinylguaiacol (4VG) production resulted in a less pronounced wheat beer aroma [8]; or artisanal bread, where decarboxylation of ferulic acid (FA) generated 4VG, an important component in its flavor profile [9]. In addition, 4VPs do not only affect the flavor of foods, but also their color, as they can react with other components in the food matrix such as anthocyanins. This interaction can result in the formation of pyranoanthocyanins [10], compounds partially responsible for imparting color to aged red wines with an enhanced resistance to bleaching [11,12]. In dairy foods, HCA decarboxylation by LAB is of special importance in cheese and yogurt, as nowadays it is not uncommon to find these products fortified with HCA-rich plant extracts [13,14,15]. Moreover, the increasing use of pigment-rich plant extracts as food colorants can also result in an increased HCA content [16,17]. Depending on the LAB strain used during production, these HCAs could be biotransformed into 4VPs, affecting the sensorial characteristics of the final product. Therefore, the study of the decarboxylating capabilities of LAB strains isolated from dairy products is a subject of increasing interest in the food industry. The most common methodologies for the screening of these decarboxylated products have been HPLC-DAD-MS [7,10,18], GC-FID [18] or GC-MS [19]. These methodologies, although precise and reliable, require expensive equipment, trained personnel, and high costs of operation. A simpler spectrophotometric method for the detection of 4VPs in a mixture was first reported using quartz cuvettes and pH 6.0 buffer [20], showing hypsochromic shifts in the λ_max_ from 285–300 nm for HCAs to 260 nm for 4VPs. This hypsochromic shift can be the basis for a modified high-throughput method that could aid in the screening and selection of LAB capable–or incapable, depending on the application–of decarboxylating HCAs. These bacteria could be used to modulate the release of compounds that affect the sensory characteristics of fermented products elaborated from HCA-rich sources.

The objective of this study was to develop a fast and simple methodology for high-throughput screening of LAB that are capable of HCA biotransformation, in order to determine the decarboxylating activity of LAB strains from the Ohio State University—Parker Endowed Chair (OSU-PECh) collection.

## 2. Results and Discussion

### 2.1. UV-Vis Spectra of HCAs, Decarboxylated HCAs and Growth Medium

**Figure 1:**
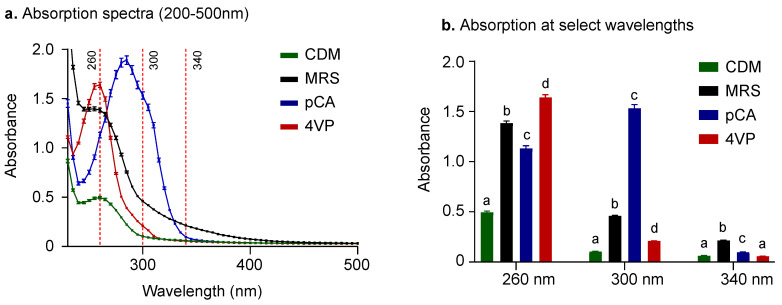
Measurements of UV-Vis light absorption at pH 7. (**a**) Absorption spectra of Man, Rogosa and Sharpe broth (MRS, black line), *p*-coumaric acid (pCA, blue line) and 4-vinylphenol (4VP, red line) in chemically defined medium (CDM) and CDM alone (green line). (**b**) Absorption at 260, 300 and 340 nm showed that MRS had a significantly higher absorption than CDM did, between 200–500 nm. Different letters indicate significant differences (*p* < 0.01). Data represent means of *n* = 3, error bars represent SD.

The first goal was to monitor the decarboxylation of HCAs using a fast, high-throughput method based on UV-Vis light absorption changes. Figure 1a shows the UV-Vis light absorption spectra of *p*-coumaric acid (pCA), 4VP, a chemically defined medium (CDM), and de Man, Rogosa and Sharpe broth (MRS) all at pH 7.0. The wavelength of maximum absorption for pCA is near 290 nm (blue line), while the one registered for 4VP is near 260 nm (red line). These results are consistent with previously reported values for chromophores with similar structural characteristics (200–280 nm for phenolic acids and 300–350 nm for HCAs) [21]. Therefore, this UV-Vis light wavelength range (200–350 nm) was a critical factor for the selection of a culture medium in which the decarboxylation experiments could be conducted, and that will allow a direct measurement of UV-Vis light absorption changes in response to structural modifications in the chromophore after incubation with LAB. Figure 1b shows the absorption of all samples at 260, 300 and 340 nm. Results showed that CDM (green bar) had a significantly lower absorption than MRS broth (black bar) at all three wavelengths analyzed (*p* < 0.01). This significantly lower absorption makes CDM an ideal medium to conduct decarboxylation experiments due to a much lower interference with the UV-Vis light absorption of the compounds of interest. The significantly lower absorption of CDM in the UV-Vis wavelength range of interest when compared to MRS broth can be attributed to a lower concentration of protein-rich components in its formulation (5 g/L in CDM and 25 g/L in MRS). Therefore, MRS broth was used for initial cell growth until the log phase was reached, and CDM was used for decarboxylation experiments and subsequent UV-Vis light absorption measurements.

### 2.2. Determination of the Optimal pH for Monitoring HCA Decarboxylation

**Figure 2:**
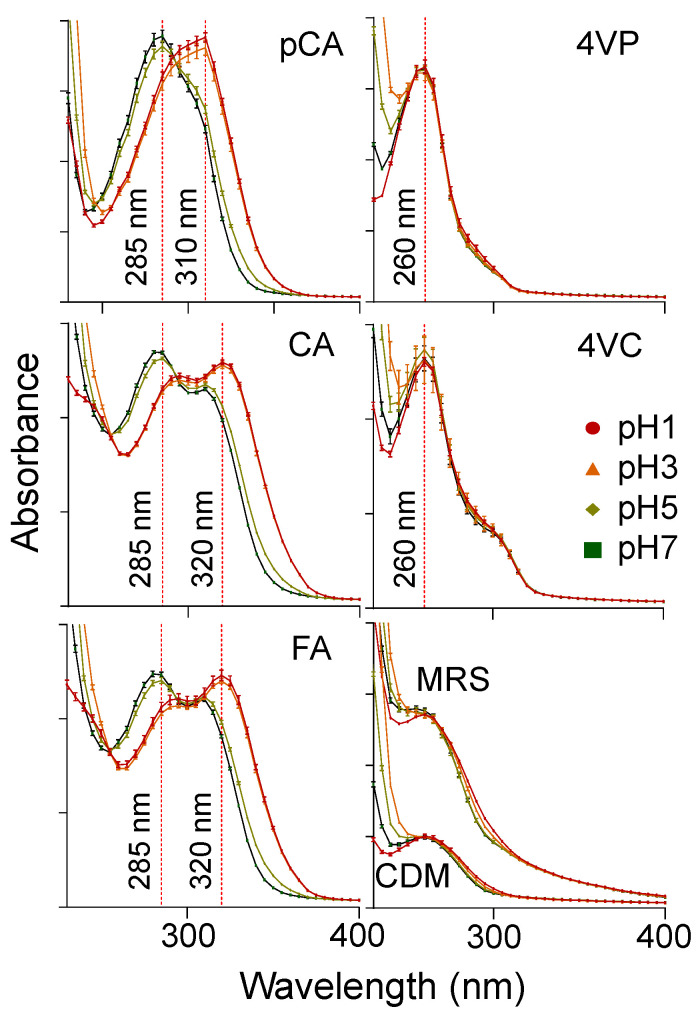
UV-Vis absorption spectra of pCA: *p*-coumaric acid, CA: caffeic acid, FA: ferulic acid, 4VP: 4-vinylphenol, 4VC: 4-vinylcatechol, MRS: De Man, Rogosa and Sharpe broth, CDM: chemically defined medium, diluted 25 times in buffers at different pH values. Bathochromic shifts were observed in the λ_230–500 max_ of HCAs when pH decreased. No changes in the λ_230–500 max_ of 4VPs were observed. Light absorption of MRS was higher than CDM at all pH values. Data represent means of *n* = 3, error bars represent SD.

After selecting an appropriate culture medium to conduct decarboxylation experiments, the next step was the determination of the optimal pH for monitoring changes in the UV-Vis light absorption in response to HCA decarboxylation by LAB. It was previously noticed that absorption increased significantly in wavelengths shorter than 230 nm, and no valuable information was obtained from that region of the spectra due to the high interference of the microplate used for assays. Therefore, subsequent experiments were focused on the light absorption changes in wavelengths longer than 230 nm. To determine the best pH for detection of changes in light absorption after incubation with LAB, the spectra of CDM containing HCAs or 4VPs were measured at pH 1.0, pH 3.0, pH 5.0 and pH 7.0. As shown in Figure 2, the λ_230–500 max_ of HCAs registered a bathochromic shift when pH decreased. At pH 1.0 (red line) and 3.0 (orange line), the λ_230–500 max_ was 309 nm for pCA, 320 nm for caffeic acid (CA), and 320 nm for FA. At pH 5.0 (light green line) and 7.0 (green line), the absorption reading was 285 nm for pCA, 284 nm for CA, and 283 nm for FA. The similarity between the λ_230–500 max_ of acidic pH (pH 1.0 and pH 3.0) and near-neutral pH (pH 5.0 and pH 7.0) can be explained by the pKa of HCAs, which were reported to be between 4.0 and 4.5 [22,23,24]. Therefore, at pH values lower than 4.0, the acid is expected to be in its undissociated form, while at pH values larger than the pKa, they are expected to be in their dissociated form [5], which ultimately changes the light absorption pattern of the chromophore. No major changes in the λ_230–500 max_ of decarboxylated products were observed in response to changes in the pH. Further measurements were conducted at pH 1.0 because it provided a more distinguishable hypsochromic shift between the λ_230–500 max_ of the HCAs and their decarboxylated products than buffers at pH 5.0 and pH 7.0. Although measurements in acidic pH (pH 1.0 and 3.0) showed similar absorbance values for the λ_230–500 max_, pH 1.0 buffer was selected due to the lower absorbance of the medium at wavelengths below 260 nm. This provided a more defined peak in the spectra, thereby allowing for a better visualization of hypsochromic changes that occur as a result of HCA decarboxylation. Moreover, the light absorption of MRS broth and CDM at different pH values were also monitored. No significant differences were found in light absorption as a response to pH changes (*p* > 0.05, data not shown); however, it is clear that the absorption of MRS broth is higher than that of CDM at all four pH values. With the data obtained, we proceeded to initially grow bacteria at their optimal pH in MRS broth, conducted decarboxylation experiments at their optimal pH in CDM, and diluted an aliquot 25 times with pH 1.0 buffer prior to measurements.

### 2.3. HPLC-DAD-ESI-MS Analysis of Decarboxylated Products

**Figure 3:**
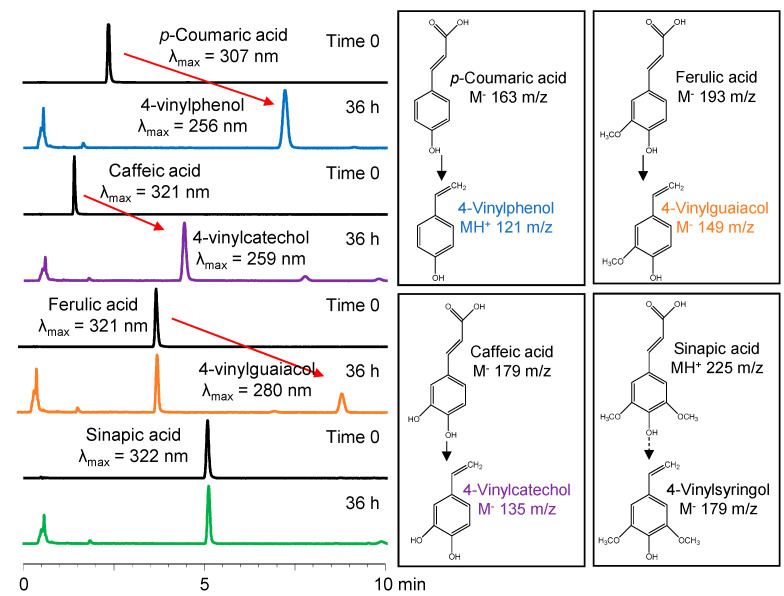
Ultra-high-pressure liquid chromatography-mass spectrometry representative chromatograms and identification of the initial hydroxycinnamic acids and their respective decarboxylated products after incubation for 36 h at 32 °C in the dark with *Lactobacillus plantarum* (OSU-PECh-BB). λ_max_: maximum absorption in the 230–500 nm range.

Results in Figure 3 showed that the degradation products from pCA, CA and FA had longer retention times, and the absorption spectra of the product peak registered by the DAD, showed a hypsochromic shift in the λ_230–500 max_ when compared to the precursor HCA (>50 nm shift), consistent with the production of decarboxylated products [20,25]. Under our experimental conditions, no sinapic acid (SA) decarboxylation was observed after incubation with LAB. This was denoted by the absence of peaks corresponding to common products from SA decarboxylation such as 4-vinylsyringol or 4-ethlysyringol. To better identify decarboxylated products, mass spectrometry analyses were conducted at time 0 and after 36 h of incubation at 32 °C in the dark. After incubation, HPLC-DAD-ESI-MS analyses confirmed the absence of pCA (M^−^ 163 *m*/*z*) and CA (M^−^ 197 *m*/*z*), and showed their transformation into their corresponding decarboxylated vinyl derivatives, 4-VP (MH^+^ 121 *m*/*z*) and 4-vinylcatechol (4VC, M^−^ 135 *m*/*z*), respectively. The product from the partial degradation of FA (M^−^ 193 *m*/*z*) was consistent with that of 4VG (M^−^ 149 *m*/*z*). Previous studies have reported the activity of a vinylphenol reductase resulting in the appearance of 4-ethylphenols derivatives from HCAs [6,20,26]. However, in this study, the HPLC-DAD-ESI-MS analyses showed no evidence of 4-ethylphenol production. The 36-h incubation at 32 °C allowed for complete decarboxylation of both pCA and CA into their 4VP derivatives with no 4-ethylphenols, making them ideal substrates for monitoring the activity of phenolic acid decarboxylase in this proposed methodology. The characteristic *m*/*z* of decarboxylated products from SA (225 *m*/*z*), 4-vinylsyringol (179 *m*/*z*) and 4-ethylsyringol (181 *m*/*z*) were also monitored, but no signal was found by the MS detector.

**Figure 4:**
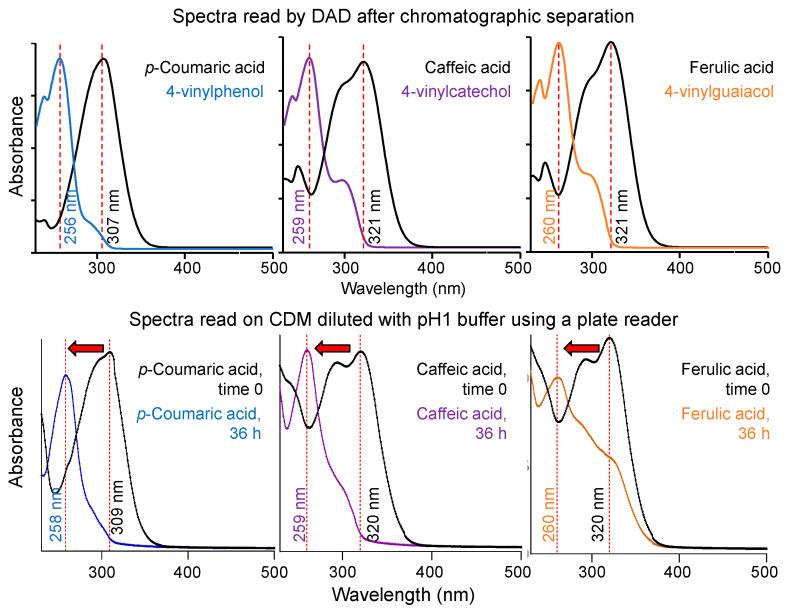
Representative UV-Vis light absorption spectra for hydroxycinnamic acids and decarboxylated products using a diode-array detector (upper) and a plate reader (lower) after incubation with *Lactobacillus plantarum* (OSU-PECh-BB). Spectra for pCA and 4VP (blue), CA and 4VC (purple), and FA and 4VG (orange). Spectra read on CDM diluted with pH 1.0 buffer using a plate reader.

In Figure 4, the absorption spectra of the individual peaks for each HCA and its respective decarboxylated derivatives (upper) are compared to the UV-Vis absorption of the bacterial supernatant measured with a plate reader directly after dilution (lower). Both methodologies were able to consistently detect hypsochromic shifts in pCA, CA and FA after incubation with LAB. These shifts occurred due to a decrease in the number of conjugated double bonds in the chromophore, as decarboxylation results in the loss of a double bond in the side chain of the HCA structure. This is consistent with previous literature showing the formation of decarboxylated products by phenolic acid decarboxylase [6,18,20]. With the plate reader, the incubated samples displayed hypsochromic shifts of 51 nm for pCA, 61 nm for CA, and 60 nm for FA. These hypsochromic shifts were similar to the ones registered by the HPLC-DAD.

### 2.4. Screening of the OSU-PECh LAB Collection for Their Ability to Decarboxylate HCAs

LAB strains isolated from dairy products from the OSU-PECh collection were screened for their ability to decarboxylate HCAs using the high-throughput UV-Vis spectrophotometric method described in Section 3.5. Results from the initial bioinformatic analysis, showed that 22 strains from a total of 137 were potentially capable of synthesizing PAD based on their reported genome annotations. *Lactobacillus plantarum* (OSU-PECh-A) was used as a positive control for PAD activity, and *Staphylococcus epidermidis* (ATCC1222) and *Lactobacillus casei* (OSU-PECh-C) were used as negative controls. A total of 24 bacterial strains were grown in CDM and incubated at 32 °C for 36 h to determine their ability to decarboxylate HCAs. Results in Table 1 show that three strains were able to decarboxylate pCA, CA and FA (*Enterococcus mundtii*, OSU-PECh-39B; *Lactobacillus plantarum*, OSU-PECh-BB and *Pediococcus pentosaceus*, OSU-PECh-27B). Six strains were able to decarboxylate pCA and CA (*Lactobacillus helveticus*, OSU-PECh-LH7; *Lactobacillus pentosus*, OSU-PECh-LP6C; *Lactobacillus plantarum*, OSU-PECh-A; *Pediococcus acidilactici*, OSU-PECh-PA3A; *Pediococcus pentosaceus*, OSU-PECh-PP13 and OSU-PECh-PP6A).Two strains were able to decarboxylate only pCA (*Lactobacillus acidophilus*, OSU-PECh-LA5 and *Lactobacillus helveticus*, OSU-PECh-LH1B); and one strain was able to decarboxylate only CA (*Lactobacillus helveticus*, OSU-PECh-LH19). No strain from the OSU-PECh collection showed the ability to decarboxylate SA under these conditions. However, this behavior was not unexpected, as literature shows that LAB may not be capable of SA decarboxylation [18]. Moreover, this selectivity against SA is apparently not unique to bacterial PAD, as yeast-derived PAD seems to have a similar behavior [27]. Although *Enterococcus mundtii*, OSU-PECh-39B; *Pediococcus pentosaceus*, OSU-PECh-27B; and *Lactobacillus plantarum*, OSU-PECh-BB were capable of decarboxylating all pCA, CA and FA; none was able to completely decarboxylate FA as they did with pCA and CA. These decarboxylation patterns for two of the three strains aforementioned are consistent with previous literature, where *Lactobacillus plantarum* [5,25,26] and *Pediococcus pentosaceous* [6] were able to metabolize pCA and CA with high efficiency, but FA only partially. However, no information on the decarboxylating capabilities of *Enterococcus mundtii* was found in published literature. This report showed the ability of the *Enterococcus mundtii* strain to decarboxylate HCAs, resulting in the production of 4VPs, strongly suggesting that this strain can in fact, synthesize PAD. This finding may promote the use of this strain for targeted bioconversion of hydroxycinnamic acids, with special interest in its capability to decarboxylate FA into 4-VG in products such as sourdough bread [28] and functional beverages obtained from lactic acid fermentation [29].

**Figure 5:**
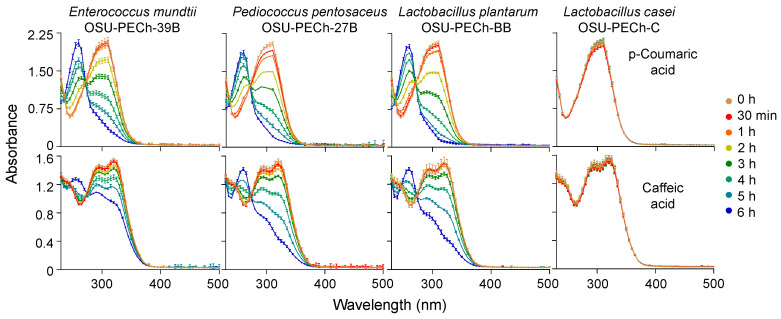
UV-Vis light absorption changes for *p*-coumaric acid (upper) and caffeic acid (lower) during incubation with *Enterococcus mundtii* (OSU-PECh-39B), *Pediococcus pentosaceus* (OSU-PECh-27B), *Lactobacillus plantarum* (OSU-PECh-BB) and *Lactobacillus casei* (OSU-PECh-C) for up to six hours at 32 °C in the dark. Data represent means of *n* = 3, bars represent SD.

Moreover, in order to investigate if the light absorption changes were time dependent, samples were taken after 30 min of incubation and every hour thereafter, and their light absorption spectra were analyzed. Figure 5 shows that the changes in the absorption spectra are in fact, time dependent and further analysis showed that pCA is decarboxylated at a faster rate than CA, with this trend being independent of the bacterial strain used. This is similar to a previous report using purified PAD, where at high concentrations of HCAs, pCA and CA were decarboxylated much faster than FA [30]. A possible explanation for this behavior may be related to the antibacterial effect of each HCA. Reports show that against *E. coli*, *S. aureus* and *B. cereus* [2], and LAB [5], pCA had a stronger antibacterial activity than a more polar CA and a less polar FA. Moreover, it was hypothesized that this stronger antibacterial activity may be related to the solubility of pCA in both aqueous and lipid phases [2]. This particular solubility pattern allows pCA to bind to the outer bacterial membrane [4], facilitating its interaction and subsequent decarboxylation by PAD.

## 3. Materials and Methods

### 3.1. Chemicals and Reagents

CA, FA, SA, dipotassium hydrogen phosphate (K_2_HPO_4_), monopotassium phosphate (KH_2_PO_4_), yeast extract, magnesium sulfate (MgSO_4_) and glucose were purchased from Sigma-Aldrich (St. Louis, MO, USA); pCA was purchased from MP Biomedicals (Solon, OH, USA); sodium chloride (NaCl) was obtained from Fisher Scientific (Pittsburgh, PA, USA); MRS lactobacilli broth was purchased from Difco Laboratories (Detroit, MI, USA). 4VP was purchased from Santa Cruz Biotechnology (Santa Cruz, CA, USA). All other reagents and solvents were of analytical grade.

### 3.2. Growth Medium Selection for HCA Decarboxylation Experiments

To develop a rapid, high-throughput spectrophotometric method, a medium with minimal light absorption interference at the wavelengths of interest had to be selected. The light absorption spectra of two growth media were compared; commercially available MRS broth, commonly used as a LAB growth medium; and CDM, designed to provide LAB with the minimum required nutrients for normal growth [31]. CDM was prepared using 0.1% K_2_HPO_4_, 0.1% KH_2_PO_4_, 0.5% yeast extract, 0.025% MgSO_4_, 0.0005% NaCl and 0.5% glucose in distilled water. Absorption spectra in the wavelengths of interest (200–500 nm) were obtained using a SpectraMax M2 plate reader (Molecular Devices, Sunnyvale, CA, USA) after diluting the growth medium 25 times with 0.1 M phosphate buffer pH 7.0.

### 3.3. Bacterial Strain Selection

LAB strains were isolated from dairy products such as milk, milk powder, buttermilk powder, cheese and yogurt [32]. Initial selection from the 137 LAB strains in the OSU-PECh collection was conducted using bioinformatic tools from the National Center for Biotechnology Information (NCBI, https://www.ncbi.nlm.nih.gov/) and genome annotations for each species [33]. Bacterial strains were selected based on their potential capability to synthesize phenolic acid decarboxylase (PAD), an enzyme responsible for metabolizing HCAs, which has been previously purified, characterized and sequenced [34]. Positive and negative controls for PAD synthesis were initially selected using the aforementioned methodology, and the PAD activity of these strains was verified using a previously published spectrophotometric method [20] and pCA as a decarboxylation substrate. Based on these preliminary studies, *Staphylococcus epidermidis* (ATCC1222) was selected as a non-LAB strain, with no PAD activity (non-LAB negative control). *Lactobacillus casei* (OSU-PECh-C) isolated from whey protein isolate produced by Hilmar Ingredients (Hilmar, CA, USA), was selected as a LAB strain showing no PAD activity (LAB negative control). *Lactobacillus plantarum* (OSU-PECh-A) isolated from natural yogurt produced by Superior Dairy (Canton, OH, USA) showing PAD activity and was selected as a LAB positive control.

### 3.4. Decarboxylation Experiments

Preparation of stock solutions and incubation in CDM was conducted using a modification of a previously reported spectrophotometric method [20]. Briefly, stock solutions of pCA, CA, FA and SA were prepared at a concentration of 5 g/L in aqueous ethanol (50% *v*/*v*). Stock solutions of HCAs were diluted in CDM for a final concentration of 500 mg/L. This allowed for a clear identification of absorption changes, as the spectrum is initially dominated by the HCA and after incubation, by the degradation product. Bacterial strains potentially capable of producing PAD were initially grown in MRS broth in sterile centrifuge tubes under microaerobic conditions, and incubated overnight at 32 °C. The culture was collected by centrifugation at 8000× *g* for 10 min at 4 °C (Centrifuge 5804R, Eppendorf, Boulder, CO, USA), the supernatant was then discarded, and pellets were washed twice with sterile saline solution (0.085% NaCl, pH 7.0). Washed pellets were reconstituted in sterile saline solution and seeded on 96-well plates with CDM containing HCAs. LAB strains were seeded at an optical density of 0.1 (O.D. 600nm) calculated using a SpectraMax M2 plate reader (Molecular Devices, Sunnyvale, CA, USA). Plates were covered with a sterile lid, sealed with parafilm to avoid evaporation, and were then incubated at 32 °C for 36 h. Each HCA used in this study was incubated individually with every LAB strain.

### 3.5. Rapid, High-Throughput Screening of Hydroxycinnamic acid Degradation by LAB Using UV-Vis Spectroscopy and Selection of Optimal pH for Measurement

Spectrophotometric analyses were used to monitor the ability of bacteria to decarboxylate HCAs. Briefly, 96-well plates were centrifuged at 4000× *g* for 30 min at room temperature (Eppendorf 5804 benchtop centrifuge, Eppendorf, Boulder, CO, USA, Eppendorf A-2-DWP microplate rotor). Supernatants were then immediately collected and frozen until further analysis. To select the best pH for monitoring absorption changes, a 10 μL aliquot of the CDM containing HCAs or their decarboxylated products, was diluted 25 times with either KCl buffer (0.25 M) at pH 1.0, citrate buffer (0.1 M) at pH 3.0 or 5.0, or phosphate buffer (0.1 M) at pH 7.0. Subsequently, the diluted aliquots were placed in a UV-transparent 96-well microplate (Thermo Scientific Nunc^®^, Wilmington, DE, USA) for the measurement of their absorbance between the range of 230–500 nm with a SpectraMax M2 plate reader (Molecular Devices, Sunnyvale, CA, USA) at 25 °C.

### 3.6. uHPLC-DAD-ESI-MS Analysis of HCAs and Degradation Products

A Nexera-i-LC-2040 3D ultra-high-pressure liquid chromatograph was used for reverse-phase chromatographic separation and UV-Vis light absorption characterization. This system consisted of 4 pumps, a refrigerated autosampler, a column oven, and a diode-array detector (DAD). Tentative identification of HCAs and their degradation products was conducted using a LCMS-8040 triple quadrupole mass spectrometer with electrospray interface (Shimadzu, Columbia, MD, USA). Briefly, 30 μL of supernatant were injected into a Restek Pinnacle DB C18 column (50 × 2.1 mm, 1.9 µm, Restek, Bellefonte, PA, USA) using a binary solvent system consisting of 0.1% aqueous formic (A) and acetonitrile (B). The elution profile was 0–10 min, 3–20% B in A, 10–11 min, 20–40% B in A, 11–13 min, 40% B (isocratic), with a flow rate of 0.3 mL/min at 40 °C. Identical ionizing conditions for the electrospray interface were used for tentative identification of degradation products: 1.5 L/min nebulizing gas, 230 °C desolvation line temperature, 300 °C heat block temperature, 15 L/min drying gas flow. MH^+^ and M^−^ of intact structures were analyzed under positive and negative mode, respectively, using the Q1 scan function from 100 to 1000 *m*/*z* and single ion monitoring function for the respective 4VPs and 4-ethylphenols at their characteristic *m*/*z*.

### 3.7. Statistical Analysis

All experiments were conducted in triplicate. Figure design, one-way ANOVA analyses and Tukey post hoc tests were conducted using GraphPad Prism 8.3.0 (GraphPad Software, San Diego, CA, USA). A value of *p* < 0.05 was considered significant.

## 4. Conclusions

The decarboxylating capabilities of a collection of LAB were successfully studied with a fast, simple, and reproducible methodology based on UV-Vis light absorption changes. Results were consistent with the formation of 4VPs, decarboxylated products from pCA, CA and FA, while no decarboxylation was observed for SA. In this study, we reported the ability of the *Enterococcus mundtii* strain to decarboxylate pCA, CA and FA; strongly suggesting that this strain is capable of synthesizing PAD. This rapid and inexpensive method has an advantage of using reagents, materials, and equipment that can be found in most laboratories. Moreover, the selection of a growth medium with low interference and the correct pH for the detection of hypsochromic changes, allow for a clearer visualization of the results. This high-throughput method will facilitate the screening of LAB strains or fermentation starters capable of enzymatic decarboxylation of different HCAs, as it does not require further preparations or extraction procedures. Furthermore, LAB grown in CDM can potentially be used for the production of 4VPs as it does not seem to promote the activity of a phenyl reductase, evidenced by the absence of 4-ethylphenol after incubation. This method can help in the selection of bacterial strains that capable or incapable, depending on the application, of decarboxylating HCAs into 4VPs, for their use in industrial fermentation processes. Ultimately, the simple, rapid method and the information conveyed in this report can promote the selection of better fermentation starters capable of targeted HCA biotransformation into valuable flavor compounds. This could result in significant quality improvements in the flavor and color of fermented products and an increased consumer acceptance. Future experiments will focus on the effect of the matrix composition and physicochemical parameters on the decarboxylating capabilities of *Lactobacillus plantarum*, *Pediococcus pentosaceus* and *Enterococcus mundtii* in different food matrices. Moreover, future studies will also focus on the expression, purification, and characterization of the PAD from *Enterococcus mundtii*.

## Figures and Tables

**Table 1:** Ability of lactic acid bacteria strains to degrade hydroxycinnamic acids. Results based on UV-Vis light absorption changes. (++): Hypsochromic changes in λ_230–500 max_ indicating complete decarboxylation of a given HCA, (+): hypsochromic changes inλ_230–500 max_ indicating partial decarboxylation of a given HCA, (−): no changes in absorption spectra. pCA: *p*-Coumaric acid, CA: caffeic acid, FA: ferulic acid, SA: sinapic acid. Results from 3 repetitions.

Lactic Acid Bacteria Strain	Accession Code	pCA	CA	FA	SA
*Enterococcus mundtii*	**OSU-PECh-39B**	(++)	(++)	(+)	(−)
*Lactobacillus acidophilus*	**OSU-PECh-LA5**	(++)	(−)	(−)	(−)
*Lactobacillus helveticus*	**OSU-PECh-25**	(−)	(−)	(−)	(−)
	**OSU-PECh-26**	(−)	(−)	(−)	(−)
	**OSU-PECh-33**	(−)	(−)	(−)	(−)
	**OSU-PECh-40**	(−)	(−)	(−)	(−)
	**OSU-PECh-57B**	(−)	(−)	(−)	(−)
	**OSU-PECh-60**	(−)	(−)	(−)	(−)
	**OSU-PECh-LH1B**	(++)	(−)	(−)	(−)
	**OSU-PECh-LH4A**	(−)	(−)	(−)	(−)
	**OSU-PECh-LH7**	(++)	(++)	(−)	(−)
	**OSU-PECh-LH15A**	(−)	(−)	(−)	(−)
	**OSU-PECh-LH19**	(−)	(++)	(−)	(−)
*Lactobacillus pentosus*	**OSU-PECh-LP6C**	(++)	(++)	(−)	(−)
*Lactobacillus plantarum*	**OSU-PECh-A**	(++)	(++)	(−)	(−)
	**OSU-PECh-BB**	(++)	(++)	(+)	(−)
*Lactobacillus rhamnosus*	**OSU-PECh-24**	(−)	(−)	(−)	(−)
*Pediococcus acidilactici*	**OSU-PECh-PA3A**	(++)	(++)	(−)	(−)
	**OSU-PECh-PAL**	(−)	(−)	(−)	(−)
*Pediococcus pentosaceus*	**OSU-PECh-27B**	(++)	(++)	(+)	(−)
	**OSU-PECh-PP6A**	(++)	(++)	(−)	(−)
	**OSU-PECh-PP13**	(++)	(++)	(−)	(−)
*Lactobacillus casei*	**OSU-PECh-C**	(−)	(−)	(−)	(−)
*Staphylococcus epidermidis*	**ATCC 1222**	(−)	(−)	(−)	(−)
